# The Assessment of Withdrawal Interval for Enrofloxacin in Yellow Catfish (*Pelteobagrus fulvidraco*) after Multiple Oral Administrations at Disparate Temperatures

**DOI:** 10.3390/ani13162568

**Published:** 2023-08-09

**Authors:** Ning Xu, Weiyu Sun, Huan Zhang, Zhi Li, Bo Cheng, Yongzhen Ding, Xiaohui Ai

**Affiliations:** 1 Yangtze River Fisheries Research Institute, Chinese Academy of Fishery Sciences, Wuhan 430223, China; xuning@yfi.ac.cn (N.X.);; 2 College of Food Science and Engineering, Wuhan Polytechnic University, Wuhan 430023, China; 3 Aquatic Products Quality and Standard Research Center, Chinese Academy of Fishery Sciences, Beijing 100141, China; 4 Agro-Environmental Protection Institute, Ministry of Agriculture and Rural Affairs, Tianjin 300191, China

**Keywords:** withdrawal time, enrofloxacin, ciprofloxacin, yellow catfish, different temperatures

## Abstract

**Simple Summary:**

Enrofloxacin (EF) is an important antibiotic in global aquaculture. Due to the abuse and the emergence of drug resistance, the excess use of EF has occurred in recent years to result in residue exceeding in the edible tissues of aquatic animals. Withdrawal time (WT) is a red line to ensure food safety for humans. However, there is no related report of WT for EF in yellow catfish (*Pelteobagrus fulvidraco*). In addition to yellow catfish being sensitive to temperature, it can alter the disposition and elimination of EF and its main metabolite in fish bodies, ciprofloxacin (CF), thereby changing the WT in tissues. Therefore, we will conduct a WT estimation of EF in yellow catfish at different temperatures to establish related regulations to help residue surveillance in fish products.

**Abstract:**

The objective of the present study was to investigate the residue depletion of EF and CF in yellow catfish to estimate its WTs in plasma and tissues after multiple oral doses for 3 days at 20 mg/kg at 15, 20, and 25 °C. Samples were collected at pre-designed time points after oral doses. A validated method was performed to quantify EF and CF in plasma and tissues by high-performance liquid chromatography. Statistical differences were conducted using one-way ANOVA analysis. According to the maximum residue limit of China and Europe considering 95% percentile with 95% confidence, the WTs were estimated to be 44, 72, 66, 99, and 95 days at 15 °C; 32, 66, 65, 86, and 73 days at 20 °C; and 32, 61, 64, 55, and 59 days at 25 °C in the plasma, muscle and skin, gill, liver, and kidney, respectively. We found that increased temperature shortened the WTs in plasma and tissues. Therefore, this study can help the risk assessment of EF in aquatic products for human health at different temperatures to avoid residue violation.

## 1. Introduction

Yellow catfish (*Pelteobagrus fulvidraco*) are extensively distributed in Asian areas. Due to their good flavor and rich nutrition, they have been greatly welcomed by consumers in China, Japan, South Korea, and southeast Asia. With the elevation of demands, the intensive cultivation method occupied the leading position for increasing the production of yellow catfish. In these environments, fish diseases easily outbreak, resulting in high mortality and huge economic losses. To date, the main pathogens are *Aeromonas veronii*, *Edwardsiella ictalurid*, and *Flavobacterium columnaris* [1,2]. To treat these diseases, many antibiotics were used in the farming of yellow catfish. The abuse and off-label use of these drugs may result in the accumulation of the drug in the fish body being over the maximum residue limits (MRLs), thereby causing issues of food safety. 

Enrofloxacin (EF) is a well-known antibiotic for opposing the above-mentioned pathogens and is ubiquitously utilized in the farming of yellow catfish. It is reported that fluoroquinolone antimicrobials can generate reactive oxygen species to cause oxidative stress [3]. If humans take animal-derived food containing an overdose of EF, it can cause mild gastrointestinal irritation or discomfort, such as headaches, dizziness, poor sleep, and other symptoms; large doses or long-term intake may lead to liver damage. To ensure food safety for humans, many countries and areas stipulated EF’s MRLs in many animals. China stipulated the residue markers of EF are the parent drug and its metabolite of ciprofloxacin (CF). According to no observed adverse effect level, its MRL was established to be 100 µg/kg in muscle, 100 µg/kg in fat, 300 µg/kg in liver, 200 µg/kg in kidney, and 100 µg/kg in milk for bovine and sheep; 100 µg/kg in muscle, 100 µg/kg in fat, 200 µg/kg in liver, and 300 µg/kg in kidney for swine, rabbits, and poultry; and 100 µg/kg in muscle and skin for fish, presented in Table 1 [4]. Japan regulated the MRLs as 50, 50, 100, and 100 µg/kg in muscle, fat, liver, kidney for cattle, pigs, and chicken; and the MRLs are 50, 50, and 100 µg/kg in edible offal for cattle, pigs, and chicken, but had no regulations for fish [5]. South Korea adopted consistent MRLs in muscle (100 µg/kg), fat (100 µg/kg), liver (300 µg/kg), and kidney (200 µg/kg) for pig, cattle, sheep, goat, rabbit, and poultry [6]. South Korea also regulated the MRLs of 100 µg/kg in crustacean muscle and fish muscle and skin [6]. Europe adopted the same MRLs as China in edible tissues for bovine, ovine, caprine, porcine, poultry, rabbits, and fish [7]. Although these MRLs have been used in real farming, the occurrence of EF residue excess often occurs in food animals, especially in fish. Therefore, the estimation of withdrawal times for EF is imperative in edible tissues of fish species.

Until now, the WT of EF has been evaluated in olive flounder (*Paralichthys olivaceus*) [8,9], rainbow trout (*Oncorhynchus mykiss*) [10], giant freshwater prawns (*Macrobrachium rosenbergii*) [11], crucian carp (*Carassius auratus gibelio*) [12], striped catfish (*Pangasianodon hypophthalmus*) [13], Nile tilapia (*Oreochromis niloticus*) [14], and northern snakehead (*Channa argus*) [15]. However, limited information on the WT of EF in yellow catfish is available. Otherwise, yellow catfish is a temperature-sensitive fish species. The changes of temperature may alter a fish’s physiological parameters that affect the disposition of the drug, thereby influencing EF’s WT in fish. So, the effect of temperature on WT cannot be neglected. In this study, the objective is to investigate the plasma and tissue depletion of EF and its metabolite, ciprofloxacin (CF), in yellow catfish at different temperatures to calculate the WTs based on statistical approaches. These will provide fundamental data of EF WT for surveillance the food safety of yellow catfish.

## 2. Materials and Methods

### 2.1. Chemicals and Reagents

Shandong Lu Kang Animal Medicine Co., Ltd. (Jining, China) provided the enrofloxacin (EF) powder with a purity equal to or more than 98.0% used for oral gavage. Analytical standards of enrofloxacin (EF) with a purity of more than 99.2% and ciprofloxacin (CF) with a purity above 95.0% used for quantitative analysis were bought from Dr. Ehrenstorfer GmbH. (Augsburg, Germany). J–T Baker (Philipsburg, USA) and Thermo Fisher (Waltham, USA) provided the reagents of methanol, water, acetonitrile, hydrochloric acid, and formic acid. The Shanghai Guoyao Company (Shanghai, China) offered anhydrous magnesium sulfate. Shanghai CNW Technologies (Shanghai, China) supported the 0.22-µm politetrafluoroetileno membranes, 10 mL and 15 mL centrifugal tubes, and 1.5 mL vials.

### 2.2. Animals 

The animal experiment was conducted in the Yaowan culturing facility of the Yangtze River Fisheries Research Institute (Jingzhou, China). All experimental protocols were approved by the Fish Ethics Committee of Yangtze River Fisheries Research Institute, Chinese Academy of Fishery Sciences, Wuhan, China. Two hundred and eighty yellow catfish (90.15 ± 10.21 g) were held in tanks (400 L per tank) receiving water (26 L/min) and were acclimatized for two weeks by being fed antibiotic-free feed. The Nutritional Research Group in our institute provided the feed consisting of 76.95% moisture, 12.79% crude protein, 3.83% crude lipid, and 3.36% ash. Three water temperatures were kept at 15.0 ± 0.5 °C, 20.0 ± 0.5 °C, and 25.0 ± 0.5 °C by an air conditioner and an aquarium heater. The parameters of water quality were determined daily and maintained to the following extent: total ammonia nitrogen concentrations ≤0.71 mg/L, dissolved oxygen concentrations at 6.3–6.8 mg/L, nitrite nitrogen concentrations < 0.067 mg/L, and pH at 6.9 ± 0.3. The blank plasma and tissues of the liver, kidney, muscle and skin, and gill were collected from ten fish not given the drug and stored at −20 °C. 

### 2.3. Repeated Oral Doses and Sample Harvest

This first step was to prepare an EF solution for oral treatment. A total of 204 mg of EF powder was accurately weighed and dissolved in 10 mL of pure water to obtain a level of 20 mg/mL. Under 15 °C, yellow catfish were administered EF daily at a dose of 20 mg/kg for 3 continuous days. The EF solution was irrigated into each fish’s stomach with a plastic tube connected to a 2.5 mL microinjector. Next, the treated fish was inspected to see whether EF solution was regurgitated from the stomach. If the fish regurgitated EF solution, it was replaced by another fish. 

After 3-day oral administrations, the operator would draw off about 1.5 mL blood from the caudal vessels of each fish (six fish for each time point) on days 1, 3, 5, 7, 14, 21, 28, 35, 42, 49, 56, 63, and 70. Blood samples were straightway centrifugated at 1500× *g* for 5 min to get plasma. Subsequently, muscle and skin, liver, kidney, and gill were also sampled from each fish. All samples were preserved at −20 °C up to measurement. The same procedures of drug treatment and sample collection were carried out under 20 and 25 °C.

### 2.4. Sample Preparation and Instrumental Analysis

The approaches of sample preparation and determination of high-performance liquid chromatography (HPLC) were consistent with our previous study. The detailed procedures were referred to the reference [16,17].

### 2.5. Method Validation

The validation method was in line with the guideline of the EU Commission Decision 2021/808/EC [18]. We investigated corresponding indices of the specificity, the limit of detection (LOD), the limit of quantification (LOQ), linearity, recovery, precision, decision limits (CC_α_), and detection capability (CC_β_). Firstly, 10 blank samples from different sources were determined by HPLC to avoid impurities from exogenous or endogenous factors in samples. If interference was found in the plot to disturb the calculation of the target drugs’ concentration, the procedures of extraction and purification should be optimized. LOD was defined as the concentration that produced a 3-time area to noise baseline. LOQ was defined as the concentration that could produce a 10-time area to noise baseline.

In this study, we adopted a matrix-fortified calibration curve to quantify target compounds. Standard solutions of EF and CF were fortified into blank samples to get different concentrations of 0.01, 0.02, 0.05, 0.1, 0.2, 1.0, and 2.0 µg/mL or µg/g. The method of sample processing was in line with the contents of Section 2.4, and then the samples were measured by HPLC. The matrix-fortified calibration curves were built by plotting real concentrations of EF and CF to the area of the corresponding peak. Simultaneously, the curve slope, intercept, and correlation coefficients were also assessed.

The recovery was assessed by determining fortified samples with three concentrations of 0.5, 1, and 1.5 times the MRL (five replicates for each concentration). Recovery rates were calculated by comparing the areas from fortified samples to the corresponding areas of real concentrations. The precision was estimated by repeatability and reproducibility. The repeatability values were analyzed as the coefficient of variation of the measured concentrations of EF or CF by analyzing the samples fortified with a standard of the analyst at three levels on the same day, with the same instrument, and by the same operator. The reproducibility values were calculated by analyzing spiked samples using the same method on separate three days.

CC_α_ was estimated by analyzing 20 blank samples spiked with EF and CF at the MRL of 0.1 µg/g in the muscle and skin of fish. CC_β_ was evaluated as CC_α_ plus 1.64 times the corresponding standard deviation. In the inedible tissues of fish, the MRL was not regulated by any country. Hence, the spiked concentrations of EF and CF in the liver, kidney, plasma, and gill were temporarily set to 0.1 µg/g or µg/mL. 

### 2.6. Statistical Analysis and WT Estimation

The concentrations of EF and CF were expressed as mean ± standard deviation. Statistical differences of EF or CF concentrations at the same time point under three temperatures were analyzed using one-way ANOVA analysis by the software of SPSS statistics 23.0. Statistical significance was confirmed by the *p*-value. If the *p*-value was equal to or less than 0.05, it was considered to be statistically significant. If the *p*-value was equal to or more than 0.05, it indicated statistically insignificant. The software of WT 1.4 was used to calculate the WTs of the target compounds in plasma and tissues, which was developed by the European Medicines Agency (EMA).

## 3. Results

### 3.1. HPLC Analysis

There is no interference existing at the time of EF and CF. EF and CF’s LOD and LOQ were 0.005 µg/mL and 0.01 µg/mL in plasma, and EF and CF’s LOD and LOQ were 0.01 µg/mg and 0.02 µg/mg in gill, liver, muscle and skin, and kidney, respectively. The plotted matrix-fortified calibration curves displayed good linearity with a coefficient of correlation of R-value ≥ 0.999. During HPLC analysis of samples, if we found the concentrations of EF or CF in some samples were more than the upper LOQ, the remaining samples were repeatedly determined after being diluted with corresponding blank samples. The average recovery values of EF were 85.73–115.21% in tissues and plasma. Their percentage-relative standard deviations for inter-day were 1.20–12.35%, and intra-day precisions were 1.72–12.77% (Table 2). The mean recovery rates of CF were 84.23–115.57% in tissues and plasma. Their inter-day relative standard deviations were 0.38–10.21%, and their intra-day precisions were 3.07–18.27% (Table 2). The CC_α_ and CC_β_ were from 107.2 to 123.5 μg/kg or μg/L.

### 3.2. Depletion Profiles of EF and CF in Plasma and Tissues

**Figure 1:**
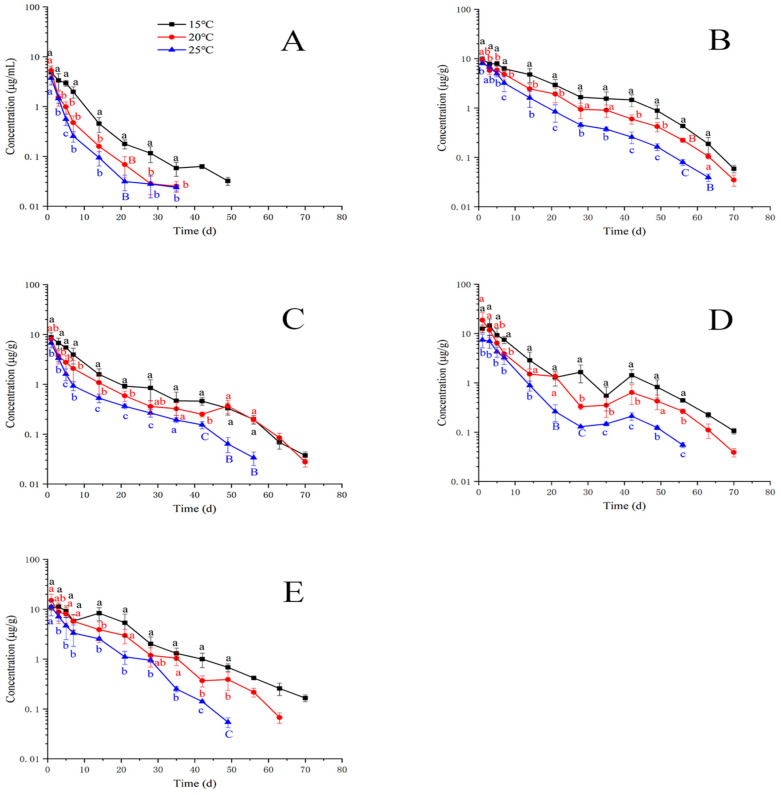
Residue depletion kinetic curves of enrofloxacin in yellow catfish (*Pelteobagrus fulvidraco*) of plasma (**A**), muscle and skin (**B**), gill (**C**), liver (**D**), and kidney (**E**) after 3-day consecutive oral administrations at a dose of 20 mg/kg at 15, 20, and 25 °C. Different letters indicate statistical significance.

**Figure 2:**
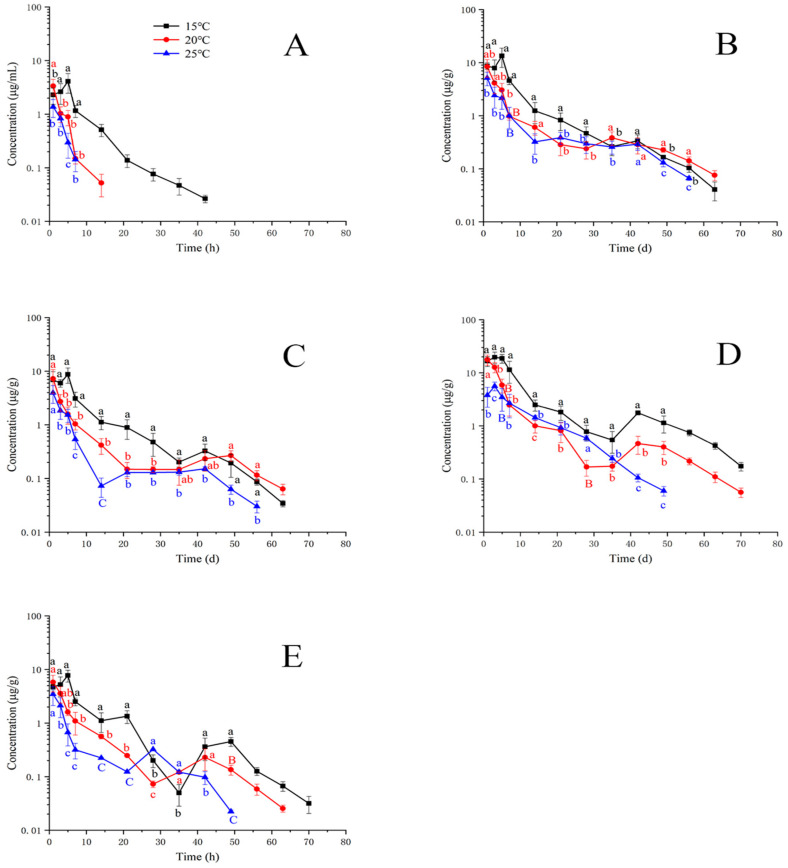
Residue depletion kinetic curves of ciprofloxacin in yellow catfish (*Pelteobagrus fulvidraco*) of plasma (**A**), muscle and skin (**B**), gill (**C**), liver (**D**), and kidney (**E**) after 3-day consecutive oral administrations at a dose of 20 mg/kg at 15, 20, and 25 °C. Different letters indicate statistical significance.

**Figure 3:**
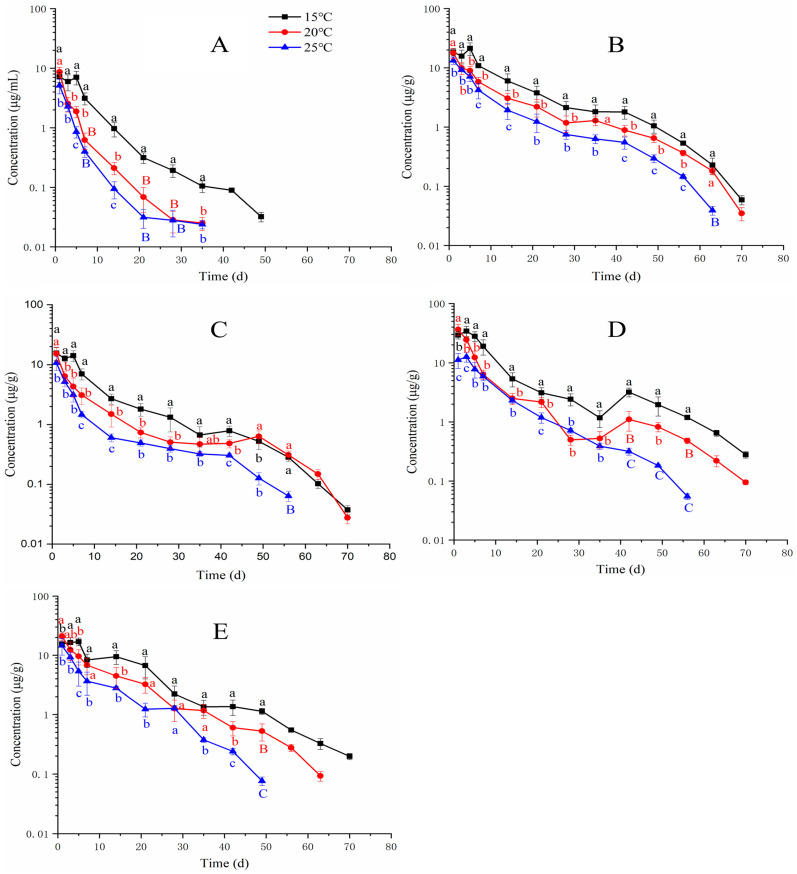
Residue depletion kinetic curves of the sum of enrofloxacin and ciprofloxacin in yellow catfish (*Pelteobagrus fulvidraco*) of plasma (**A**), muscle and skin (**B**), gill (**C**), liver (**D**), and kidney (**E**) after 3-day consecutive oral administrations at a dose of 20 mg/kg at 15, 20, and 25 °C. Different letters indicate statistical significance.

Figure 1 and Figure 2 exhibit the concentration–time curves of EF and CF in plasma and tissues (liver, kidney, muscle and skin, and gill) at different temperatures. The combined concentration–time profiles of EF and CF are shown in Figure 3. As shown in Figure 1A, the concentration of EF in plasma was above the LOQ until 49 days after oral dosing at 15 °C, and its value was higher than the LOQ until 35 days after oral dosing at 20 and 25 °C. Overall, the concentrations of EF at 15 °C were significantly higher than those at 20 and 25 °C. In the muscle and skin, the EF residue concentrations at 15 °C were significantly higher than those at 20 and 25 °C, especially concentrations at 56 and 63 days which were significantly higher at 15, 20, and 25 °C. In the gill, the differences in EF concentration were insignificant after 49 days at 15 and 20 °C. The EF concentrations at 42, 49, and 56 days under 25 °C were significantly lower than those at 15 and 20 °C. In the liver, differences in EF concentrations at 21 and 28 days at 25 °C were significantly lower than those under 15 and 20 °C. The EF concentrations at 35 days were lower than those at 42 days at the three temperatures. After 42 days, the EF concentrations gradually decreased over time. In the kidney, most EF concentrations at 25 °C were significantly different from those at 15 and 20 °C. The EF levels after 49 days were lower than LOQ. Table 3 shows the elimination half-lives (T_1/2_) of EF in plasma and tissues. The results showed that the values of T_1/2_ in plasma were the lowest compared to those in tissues at the three temperatures. With the increase in temperature, the T_1/2_ values were successively decreased in tissues but not in plasma.

Figure 2 shows the concentration–time profiles of CF in plasma and tissues at 15, 20, and 25 °C. Due to CF being a metabolite of EF, the maximum concentration was not all presented at the first sampling time point. For example, the peak concentration of CF in the muscle and skin was displayed at 5 days after oral treatment at 15 °C. Figure 2A exhibits that the concentration of CF was detectable up to 42 days at 15 °C, up to 14 days at 20 °C, and up to 7 days at 25 °C. The concentrations of CF at 15 °C at 3, 5, and 7 days were significantly higher than those at 20 and 25 °C. Figure 2B shows the concentrations of CF at 15 °C were significantly thicker compared to those at 20 and 25 °C before 42 days. At 42 days, their differences are insignificant among the three temperatures. After 49 days, the concentrations of CF at 20 °C were higher than those at 15 and 25 °C. Figure 2C shows that the concentrations of CF at 25 °C were less than those at 15 and 20 °C. After 49 days, the concentrations of CF at 20 °C were higher than those at 15 and 25 °C. Figure 2D shows that the concentrations of CF at 15 °C in the liver were significantly higher than those at 20 and 25 °C. However, the concentrations of CF at 7–35 days at 20 °C were lower than those at 25 °C. Figure 2E shows that most concentrations of CF at 15 °C were higher than those at 20 and 25 °C, except for 28 and 35 days. Table 4 lists the T_1/2_ of CF in plasma and tissues. Compared to T_1/2_ in tissues, the T_1/2_ in plasma was the shortest at the same temperature.

Figure 3 shows the combined concentration–time profile of EF and CF in plasma and tissues at 15, 20, and 25 °C. In plasma, the total residues of EF and CF at 20 and 25 °C were significantly less than those at 15 °C from 3 to 35 days after repeated oral doses. In the muscle and skin, the total residue profile showed that the concentrations at 20 and 25 °C were also significantly less than those at 15 °C. In the gill, the total residue at 20 °C presented an increasing trend from 35 to 42 days after oral doses. Statistical analysis showed that the concentration at 20 °C at 42 days was significantly higher than those at 15 and 25 °C. In the liver, from 1 to 35 days after drug treatment, a decreased trend was presented for total residues at the three temperatures. However, concentrations suddenly ascended at 42 days and then reduced. In the kidney, the order of total residues at each time point was 15 ˃ 20 ˃ 25 °C. Table 5 shows the T _1/2_ of total residues in plasma and tissues. At 15 °C, the order of T_1/2_ was liver ˃ kidney ˃ muscle and skin ˃ gill ˃ plasma. At 20 °C, the order of T_1/2_ was gill ˃ liver ˃ muscle and skin ˃ kidney ˃ plasma. At 25 °C, the order of T_1/2_ was gill ˃ muscle and skin ˃ liver ˃ kidney ˃ plasma.

### 3.3. The Estimation of Withdrawal Times

**Figure 4:**
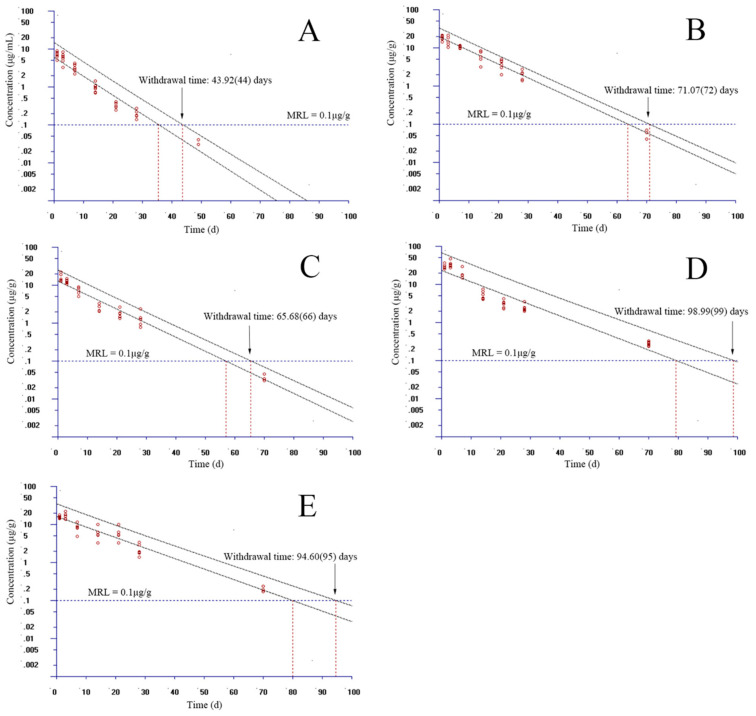
Withdrawal intervals of enrofloxacin in yellow catfish (*Pelteobagrus fulvidraco*) of plasma (**A**), muscle and skin (**B**), gill (**C**), liver (**D**), and kidney (**E**) after 3-day consecutive oral administrations at a dose of 20 mg/kg at 15 °C based on the standard of China. MRL: maximum residue limit for enrofloxacin from the Ministry of Agriculture and Rural Affairs of the People’s Republic of China.

**Figure 5:**
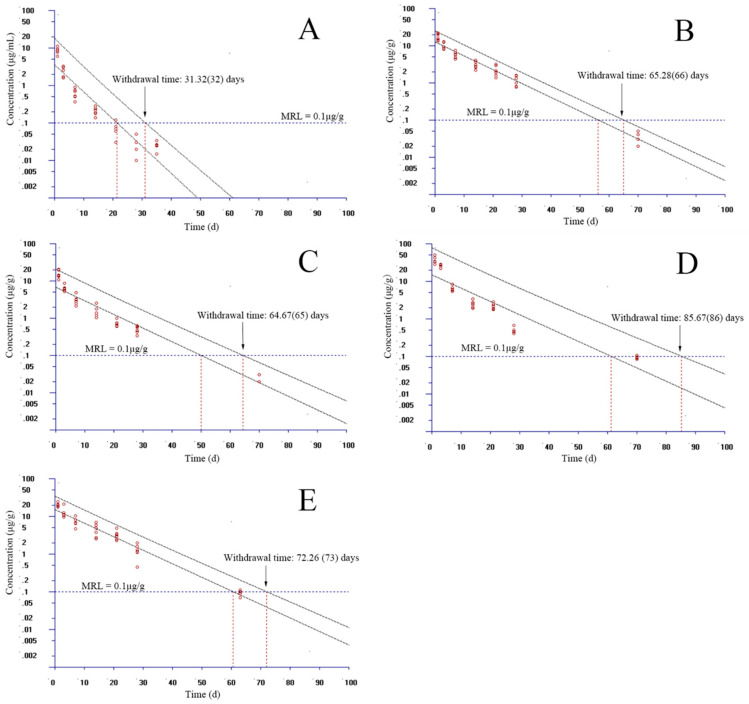
Withdrawal intervals of enrofloxacin in yellow catfish (*Pelteobagrus fulvidraco*) of plasma (**A**), muscle and skin (**B**), gill (**C**), liver (**D**), and kidney (**E**) after 3-day consecutive oral administrations at a dose of 20 mg/kg at 20 °C based on the standard of China. MRL: maximum residue limit for enrofloxacin from the Ministry of Agriculture and Rural Affairs of the People’s Republic of China.

**Figure 6:**
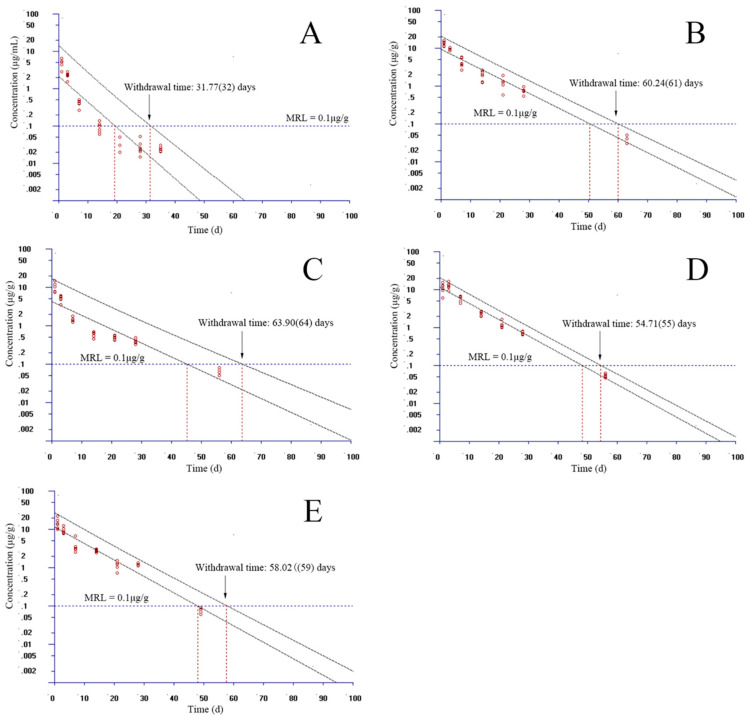
Withdrawal intervals of enrofloxacin in yellow catfish (*Pelteobagrus fulvidraco*) of plasma (**A**), muscle and skin (**B**), gill (**C**), liver (**D**), and kidney (**E**) after 3-day consecutive oral administrations at a dose of 20 mg/kg at 25 °C based on the standard of China. MRL: maximum residue limit for enrofloxacin from the Ministry of Agriculture and Rural Affairs of the People’s Republic of China.

In the standard of GB 31650-2019 in China, the residue marker of EF is the parent drug and CF. The MRL was stipulated to be 0.1 µg/g in the muscle and skin of fish. However, the MRLs in the liver, kidney, and other tissues were not regulated. In the present study, the MRL of EF in the plasma, liver, kidney, and gill borrowed the value from the muscle and skin. Given that the WT 1.4 software allows for only seven time points to be considered, we selected the proper time points to estimate the WT at each temperature. At 15 °C, time points were selected as 1, 3, 7, 14, 21, 28, and 49 days in plasma, and 1, 3, 7, 14, 21, 28, and 70 days in muscle and skin, gill, liver, and kidney, respectively. Considering the 95% percentile with a 95% confidence, the WTs were estimated to be 44, 72, 66, 99, and 95 days in the plasma, muscle and skin, gill, liver, and kidney, respectively (Figure 4). At 20 °C, time points were chosen as 1, 3, 7, 14, 21, 28, and 35 days in plasma; 1, 3, 7, 14, 21, 28, and 63 days in the kidney; and 1, 3, 7, 14, 21, 28, and 70 days in the muscle and skin, gill, liver, respectively. Considering the same percentile with confidence, the WTs were assessed to be 32, 66, 65, 86, and 73 days in the plasma, muscle and skin, gill, liver, and kidney, respectively (Figure 5). At 25 °C, we selected 1, 3, 7, 14, 21, 28, and 35 days for plasma; 1, 3, 7, 14, 21, 28, and 63 days for the muscle and skin; 1, 3, 7, 14, 21, 28, and 49 days for the kidney; and 1, 3, 7, 14, 21, 28, and 56 days for the gill and liver. The WTs were evaluated to be 32, 61, 64, 55, and 59 days in the plasma, muscle and skin, gill, liver, and kidney, respectively (Figure 6).

## 4. Discussion

The present study examined the plasma and tissue depletion of EF and CF and estimated the WTs in yellow catfish after 3-day consecutive oral doses at 20 mg/kg at the different temperatures of 15, 20, and 25 °C, respectively. HPLC was used to analyze residue samples of yellow catfish to estimate the WTs of EF. The LOQ of EF and CF were 0.01 µg/mL in plasma, and the LOQ of EF and CF were 0.01 µg/g and 0.02 µg/g in the muscle and skin, gill, liver, and kidney, respectively [12]. They are far lower than the MRL of 0.1 µg/g. Moreover, this method was validated using the guideline of the EU Commission Decision 2021/808/EC [18,19,20]. Hence, this quantitative method of EF and CF is sufficient for the requirement of WT estimation. We found that the concentrations of EF and CF in plasma were lower than those in tissues at any temperature, suggesting that EF and CF have an accumulation trend in tissues. The concentrations of EF in the liver, kidney, and muscle were higher than those in the gill and plasma. The concentrations of EF in the liver before 5 days at 15 and 20 °C were higher than those in the kidney, but the concentrations after 5 days were lower than those in the kidney, suggesting that the EF in the liver before 5 days was in the absorption and metabolism phase and then, after 5 days, was in the excretion phase. When it was in the excretion phase, the concentration of EF in the kidney were higher than that in the liver, indicating that EF was mainly excreted by the kidney. CF is a metabolite of EF whose concentrations in the liver were the highest compared to those in other tissues, demonstrating that CF was generated by the metabolism of the liver in yellow catfish [21]. At 15 °C, the concentrations of CF presented two peaks at 5 and 42 days after 3-day consecutive oral doses, but this phenomenon was not exhibited at 20 and 25 °C. The detailed reason was unknown. We speculated that the temperature-dependent absorption, metabolism, and excretion of EF and CF might lead to this phenomenon. 

The metabolism of EF has been extensively evaluated in rats, cattle, pigs, and poultry by oral administration of ^14^C-enrofloxacin [22]. In rats, the main metabolites of EF were CF, EF glucuronide, and an unidentified metabolite in urine and bile. In pigs, the major residues were the parent drug and CF, which account for 80% of the total residues in the liver, kidney, muscle, and fat. In cattle, EF and CF were also the main residues in the edible tissues. The difference was that the amount of CF was similar to that of the parent drug. Studies of the unlabeled drug found similar results as well. However, EF and CF were the major residues in the liver, but only EF was presented in muscle and skin in poultry [22]. From these results, CF is the main metabolite of EF in land animals. Although no study has been conducted on fish using ^14^C-enrofloxacin, many studies adopted an unlabeled method [12,23,24]. Meanwhile, CF was determined to be the major metabolite of EF in fish. The residue marker was stipulated to be the parent drug and CF in fish by China, Europe, Japan, South Korea, and so on. In the present study, the main residues were EF and CF in plasma and the edible tissues of yellow catfish. EF and CF were detectable for a long time in tissues after multiple oral doses. EF could be quantified in the muscle and skin, liver, and gill at 15 and 20 °C up to 70 days after 3-day consecutive oral administration at a dose of 20 mg/kg, and it can be detectable in the muscle and skin at 25 °C up to 63 days at the same administration. CF was measurable in the liver and kidney at 15 and 20 °C up to 63 days after oral doses. Therefore, EF and CF were accumulated in tissues of yellow catfish, especially in cold water, which needs sufficient WT to ensure the residue concentration falls below the MRL of 100 µg/kg.

Increased temperature shortened the T_1/2_ of EF, CF, and combined EF and CF. Compared to the values at 15 °C, T_1/2_ of combined EF and CF was decreased by 26.37%, 8.93%, 33.71%, and 33.33% in the plasma, muscle and skin, liver, and kidney, respectively. This finding was consistent with the T_1/2_ study on the same drug and other drugs [23,25,26,27]. A study reported that the T_1/2_ of EF at 10 °C was 136.59 h, which was decreased to 98.63 h at 25 °C in plasma after oral administration at a dose of 10 mg/kg, which is close to the calculated values at 25 °C in the present study [28]. For florfenicol, the T_1/2_ in the plasma, liver, kidney, and muscle and skin at 10 °C was more than two-fold in the corresponding plasma and tissues at 25 °C after 5-day consecutive oral administration of FF at a dose of 10 mg/kg [23]. These results demonstrated that warmer temperatures also accelerated the excretion rate of the aquatic drug because fish are heterothermic animals [26,29,30]. Their physiological activity was sensitive to temperature, thereby changing the pharmacological parameters of aquatic drugs to shift the WTs in tissues.

In the present study, the WTs were 44, 72, 66, 99, and 95 days; 32, 66, 65, 86, and 73 days; and 32, 61, 64, 55, and 59 days in the plasma, muscle and skin, gill, liver, and kidney at 15, 20, and 25 °C, respectively. The total trends of WT in plasma and tissues were decreased with the rise of temperature. This finding was consistent with the previous study in florfenicol at different temperatures in crucian carp. Many WT studies have reported on many fish species at one temperature. Lucchetti, Fabrizi, Guandalini, Podesta, Marvasi, Zaghini, and Coni [10] found that the WT of EF was 39 days in the muscle and skin of rainbow trout (*Oncorhynchus mykiss*) at a real fish farm via medication-feed-given EF at a dose of 10 mg/kg for 5 days at 13 °C. The WT was calculated to be 11 days in the muscle of freshwater prawns (*Macrobarachium rosenbergii*) after the treatment of medication feed containing an EF concentration of 5 g/kg at a rate of 1% of total body weight (being equal to 10 mg/kg BW of pure EF) twice a day for 5 consecutive days [11]. Shan, Huang, Zheng, Yin, Zhu, Ma, Zhou, Xie, Li, Liu, and Wang [12] reported that the WT of EF was 32 days in the muscle and skin of crucian carp (*Carassius auratus gibelio*) following multiple oral gavages at 20 mg/kg for 5 days at 28 °C. When striped catfish (*Pangasianodon hypophthalmus*) were treated daily with medicated feed at 10 mg/kg of EF for five consecutive days following farmer procedures, the WT was estimated to be 45 days [13]. In northern snakehead (*Channa argus*), the WT was estimated to be 18 days in the muscle and skin at 25 °C when given EF at a dose of 10 mg/kg daily via oral administration for 5 days [15]. Overall, our estimated WT of EF was longer than the values reported in previous studies. A possible reason may be that we re-calibrated the content of commercial EF in line with the analytical standard of EF. Other reasons may be different dosages, oral rates, the methods of the given drug, temperatures, fish species, and environmental factors.

## 5. Conclusions

The present study conducted the residue depletion of EF and CF in yellow catfish after 3-day oral treatment of EF at a dose of 20 mg/kg at three temperatures to estimate its WT in plasma and edible tissues. According to the MRL of China and Europe considering 95% percentile with 95% confidence, the WTs were estimated to be 44, 72, 66, 99, and 95 days at 15 °C; 32, 66, 65, 86, and 73 days at 20 °C; and 32, 61, 64, 55, and 59 days at 25 °C in the plasma, muscle and skin, gill, liver, and kidney, respectively. With the increase of temperature, the WT was obviously shortened in plasma and tissues. Therefore, different WTs should be adopted at different temperatures.

## Figures and Tables

**Table 1:** Maximum residue limit of enrofloxacin in various countries.

Countries	Maximum Residue Limit in Fish	Maximum Residue Limit in Other Animals
China	Fish: muscle and skin (100 µg/kg)	Bovine and sheep: muscle (100 µg/kg), fat (100 µg/kg), liver (300 µg/kg), kidney (200 µg/kg), and milk (100 µg/kg). Swine, rabbits, poultry: muscle (100 µg/kg), fat (100 µg/kg), liver (200 µg/kg), and kidney (300 µg/kg).
Europe	Fish: muscle and skin (100 µg/kg)	Bovine and sheep: muscle (100 µg/kg), fat (100 µg/kg), liver (300 µg/kg), kidney (200 µg/kg), and milk (100 µg/kg). Swine, rabbit, poultry: muscle (100 µg/kg), fat (100 µg/kg), liver (200 µg/kg), and kidney (300 µg/kg).
Japan	-	Cattle, pigs, and chickens: muscle (50 µg/kg), fat (50 µg/kg), liver (100 µg/kg), and kidney (100 µg/kg). Cattle, pigs, and chickens: 50 µg/kg, 50 µg/kg, and 100 µg/kg in edible offal.
South Korea	Crustacean: muscle (100 µg/kg); fish: muscle and skin (100 µg/kg)	Pig, cattle, sheep, goat, rabbit, and poultry: muscle (100 µg/kg), fat (100 µg/kg), liver (300 µg/kg), and kidney (200 µg/kg).

Note: -, not available.

**Table 2:** Accuracy and precision of the method for enrofloxacin and ciprofloxacin in spiked plasma or tissues in yellow catfish (*Pelteobagrus fulvidraco*).

Drug	Plasma or Tissues	Spiked Concentration (μg/g or μg/mL)	Recovery/%	Intra-Day RSD (%)	Inter-Day RSD/%
Enrofloxacin	Plasma	0.02	107.33 ± 4.21	1.78	9.45
		0.1	92.33 ± 5.21	2.37	7.02
		1	86.27 ± 2.07	3.48	4.21
	Muscle and skin	0.02	102.33 ± 6.77	3.08	8.01
		0.1	87.21 ± 1.88	2.24	4.01
		1	85.73 ± 1.99	3.22	4.34
	Gill	0.02	115.21 ± 7.07	5.32	8.21
		0.1	90.58 ± 3.29	2.75	3.92
		1	87.25 ± 1.07	2.33	4.25
	Liver	0.02	104.55 ± 13.47	12.35	12.77
		0.1	89.47 ± 5.28	4.37	10.59
		1	88.59 ± 4.25	3.09	1.72
	Kidney	0.02	105.88 ± 6.37	2.51	6.29
		0.1	108.59 ± 9.21	1.20	6.33
		1	86.22 ± 1.77	2.38	3.97
Ciprofloxacin	Plasma	0.02	105.89 ± 6.12	3.77	6.38
		0.1	96.58 ± 1.59	2.69	3.07
		1	86.43 ± 1.22	3.29	4.28
	Muscle and skin	0.02	106.77 ± 5.68	10.21	9.35
		0.1	108.59 ± 2.37	4.67	5.84
		1	84.23 ± 2.88	3.01	3.82
	Gill	0.02	115.57 ± 5.37	4.01	5.32
		0.1	104.86 ± 4.35	3.85	4.27
		1	88.59 ± 2.57	1.08	3.09
	Liver	0.02	104.25 ± 8.02	8.59	18.27
		0.1	101.77± 3.92	1.55	1.98
		1	85.39 ± 1.07	0.38	3.79
	Kidney	0.02	105.32 ± 8.22	4.09	8.67
		0.1	95.22 ± 4.33	5.21	7.08
		1	86.55 ± 1.68	2.31	6.32

**Table 3:** Elimination half-lives of enrofloxacin in yellow catfish (*Pelteobagrus fulvidraco*) of plasma, muscle and skin, gill, liver, and kidney after 3-day consecutive oral administrations at a dose of 20 mg/kg at 15, 20, and 25 °C.

Temperatures (°C)	Elimination Half-Lives (Days)				
	Plasma	Muscle and Skin	Gill	Liver	Kidney
15	6.48	11.00	9.90	11.18	11.18
20	4.65	9.90	11.00	9.63	9.00
25	4.91	8.56	8.77	7.97	6.86

**Table 4:** Elimination half-life of ciprofloxacin in yellow catfish (*Pelteobagrus fulvidraco*) of plasma, muscle and skin, gill, liver, and kidney after 3-day consecutive oral administrations at a dose of 20 mg/kg at 15, 20, and 25 °C.

Temperatures (°C)	Elimination Half-Lives (Days)				
	Plasma	Muscle and Skin	Gill	Liver	Kidney
15	5.59	8.25	8.56	11.75	9.90
20	2.19	11.75	12.38	10.19	9.76
25	1.77	11.18	10.19	7.62	9.12

**Table 5:** Elimination half-life of the sum of enrofloxacin and ciprofloxacin in yellow catfish (*Pelteobagrus fulvidraco*) of plasma, muscle and skin, gill, liver, and kidney after 3-day consecutive oral administrations at a dose of 20 mg/kg at 15, 20, and 25 °C.

Temperatures (°C)	Elimination Half-Lives (Days)				
	Plasma	Muscle and Skin	Gill	Liver	Kidney
15	6.03	9.63	9.00	11.36	10.83
20	4.15	9.76	10.66	9.90	9.12
25	4.44	8.77	9.24	7.53	7.22

## Data Availability

Not applicable.

## Abbreviations

CC_α_	decision limits
CC_β_	detection capability
EF	enrofloxacin
FF	florfenicol
LOD	the limit of detection
LOQ	the limit of quantification
MRL	maximum residue limit
T_1/2_	elimination half-life
HPLC	high-performance liquid chromatography
WT	withdrawal time
